# Kidney disease and cardiac surgery: marker, mediator, or both?

**DOI:** 10.1016/j.bjao.2025.100507

**Published:** 2025-12-04

**Authors:** Abraham H. Hulst, Fabrizio Monaco

**Affiliations:** 1 Department of Anaesthesiology, Amsterdam UMC, University of Amsterdam, Amsterdam, The Netherlands; 2 Department of Cardiothoracic and Vascular Anesthesia, IRCCS Ospedale San Raffaele, Milan, Italy; 3 Division of Cardiacthoracic and Vascular Anesthesia, IRCCS Azienda Ospedaliero-Universitaria di Bologna, S. Orsola Hospital, University of Bologna, Bologna, Italy; 4 Department of Medical and Surgical Sciences, DIMEC, University of Bologna, Bologna, Italy

**Keywords:** acute kidney injury, cardiac surgery, chronic kidney disease, renal failure, risk predictor

## Abstract

Cardiac surgery places an enormous burden on the kidneys, and therefore, acute kidney injury (AKI) is a common postoperative complication. Pre-existing chronic kidney disease (CKD) is a strong predictor of cardiac surgery-associated AKI, in addition to the evidence of a bidirectional interaction where AKI also accelerates the progression of CKD and increases the risk of renal failure. While observational research links kidney dysfunction to prolonged hospital stays, morbidity, and mortality, the question remains whether AKI is a modifiable mediator or merely a perioperative risk predictor. Larger analyses, including that of Bille and colleagues, indicate that AKI drives the acceleration of CKD. These findings provide further evidence for systematic follow-up and early implementation of kidney-protective measures to optimise long-term renal function after cardiac surgery.

With >1.5 million procedures annually, cardiac surgery remains one of the most common high-impact surgical interventions.^1^ Unlike most surgical procedures, cardiac surgery represents a profound physiological stress affecting all vital organs, of which the kidneys are especially sensitive. As a result, acute kidney injury (AKI) is one of the most common postoperative complications. While heavily dependent on data quality, patient characteristics, and diagnostic criteria, incidences up to 70% have been reported.^2^

Reduced renal function or chronic kidney disease (CKD) is one of the strongest predictors of cardiac surgery-associated AKI (CSA-AKI) before operation.^3^^,^^4^ In methodologically rigorous analyses, Bille and colleagues^5^ examined >27 000 cardiac surgery cases, with complete mortality data and a median follow-up duration of 7 yr (range 0–24 yr). By using competing-risk modelling, they provided a particularly robust assessment of long-term renal outcomes, effectively mitigating the survivorship bias that has constrained previous studies. Their findings offer compelling evidence that the relationship between CKD and CSA-AKI is bidirectional, with AKI itself predicting subsequent kidney disease progression and failure.

Observational studies have consistently shown that kidney dysfunction, either in the form of pre-existing CKD or perioperative AKI, is associated with prolonged hospitalisation, cardiovascular events, and mortality. Beyond this, however, a fundamental question remains: are kidney diseases merely markers identifying high-risk patients, or are they modifiable mediators that causally contribute to adverse outcomes? This distinction carries profound clinical implications. If kidney dysfunction simply reflects underlying patient frailty, comorbidity, or disease severity, then efforts to improve kidney function may fail to improve outcomes. Conversely, if kidney injury directly drives complications, then interventions aimed at preserving or restoring the kidney could meaningfully improve recovery and long-term prognosis after surgery.

Improving kidney function before cardiac surgery is rarely feasible. Patients typically present with longstanding CKD that has developed over years or decades. Short-term interventions optimising volume status, holding nephrotoxic medications, and correcting metabolic derangements may marginally improve kidney function but cannot reverse chronic structural damage. Thus, in most patients, preoperative kidney function likely represents a ‘fixed’ risk factor in patients presenting for surgery.

Postoperative AKI initially appeared more amenable to prevention, prompting decades of research into pharmacological and procedural interventions. Yet this effort has yielded a complex pattern of results that challenges our understanding of AKI as a modifiable mediator of adverse outcomes.

Until now, multiple, initially promising interventions have failed to reduce CSA-AKI in adequately powered trials. Fenoldopam, a selective dopamine-1 receptor agonist, increases renal blood flow but only modestly improves urine output and creatinine clearance, without a meaningful reduction in AKI, need for renal replacement therapy, or hospital length of stay.^6^ Similarly, atrial natriuretic peptide was tested for its natriuretic and vasodilatory properties, but it did not reduce the incidence of renal dysfunction or mortality.^7^ N-acetylcysteine has been tested after positive results in contrast-induced nephropathy. In the cardiac surgery setting, multiple trials failed to show a reduction in AKI, dialysis requirement, or mortality.^8^

While some recent studies reported on interventions effective in reducing AKI, many failed to improve other meaningful outcomes. This further underscores the question of whether AKI genuinely mediates adverse outcomes or merely reflects underlying illness. Amino acid supplementation was proposed to provide metabolic substrate to energy-depleted tubular cells while maintaining renal blood flow through vasodilatory effects. While a large multicentre trial demonstrated a 15% reduction in CSA-AKI, it did not show improvements in dialysis requirement, hospital length of stay, or mortality.^9^ It is difficult to interpret whether amino acids truly prevent injurious kidney damage or induce a physiological variation in creatinine. The PrevAKI strategy ^10^^,^^11^ uses urinary biomarkers (TIMP-2•IGFBP7) to risk-stratify patients and trigger implementation of the Kidney Disease: Improving Global Outcomes (KDIGO) AKI prevention bundle. This strategy resulted in a 10% absolute reduction in moderate-severe AKI in a multicentre randomised trial of 278 high-risk cardiac surgery patients. Once again, it did not observe differences in other outcomes.^11^

In contrast, a small number of AKI prevention trials also showed benefits on other outcomes. The VANCS trial investigated low-dose vasopressin *vs* norepinephrine for blood pressure support during and after cardiac surgery. Beyond the primary haemodynamic endpoints, vasopressin not only reduced the incidence of AKI but also decreased the need for renal replacement therapy and shortened lengths of stay in ICU or hospital.^12^ A large-scale vasopressin trial is currently underway to definitively establish whether this approach improves kidney outcomes and survival in cardiac surgery patients.^13^

Against this background of uncertainty, the comprehensive analysis by Bille and colleagues provides several important insights that help advance our understanding of kidney disease as both a marker and a possible mediator in the context of cardiac surgery.

First, their data confirm a clear dose-response relationship between baseline CKD severity and postoperative AKI risk. AKI occurred in 29% of patients with normal kidney function (stages G1–2), increasing progressively to 80% in stage G5. Moreover, the severity of AKI increased with worsening baseline kidney function: among patients who developed AKI, stage 3 AKI occurred in only 10.6% of those with normal baseline function but in 55.5% of stage G4 patients. This graded relationship between baseline kidney disease severity and both AKI incidence and intensity suggests that pre-existing kidney damage increases vulnerability to subsequent injury.

Second, and perhaps most compellingly, the authors demonstrate that postoperative AKI substantially increases the risk of long-term CKD progression. Among patients with baseline CKD stages G3a–G4, those who developed AKI showed markedly elevated 3-yr risks compared with those without AKI. Kidney failure increased nearly five-fold (from 1.2% to 5.6%), CKD stage progression more than doubled (from 10.1% to 23.2%), and rapid progression increased by one-third (from 24.7% to 32.4%). Critically, patients who did not develop AKI showed progression rates comparable with population-based CKD cohorts, suggesting that the excess burden of kidney disease progression in cardiac surgery patients is largely AKI-mediated.

Third, the pattern of progression over time provides mechanistic insights. The clustering of progression events in the first postoperative year, particularly in AKI patients, suggests that perioperative kidney injury triggers processes that result in accelerated loss rather than simply identifying patients destined to progress irrespective. The observation of CKD progression primarily in the first year after surgery creates a window of opportunity for kidney-specific observation and intervention. This period provides an opportunity for the initiation of structured follow-up programs and a rationale for the early introduction of effective and novel kidney-protective therapies such as RAAS blockade and SGLT2 inhibition.^14^^,^^15^

In the absence of adequate evidence from prospective randomised studies with a long-term follow-up, the Bradford Hill criteria offer a constructive framework to evaluate the likelihood of causality to explain correlated findings from observational studies. Notable criteria found in the study by Bille and colleagues include: the strength of association between AKI and CKD progression, with relative risks exceeding four-fold; the dose-response relationship is evident in the graded association between baseline CKD severity, AKI severity, and progression risk; they demonstrated consistency across multiple outcome measures (e.g. rapid progression, chronic kidney failure); temporality is clearly established, with AKI preceding rather than after CKD progression; biological plausibility is provided by extensive mechanistic studies demonstrating AKI triggering maladaptive repair, inflammation, fibrosis, and microvascular loss.^16^

The study findings echo and extend previous investigations. Ishani and colleagues^17^ analysed older individuals and found that those with AKI, particularly in combination with CKD, were at significantly increased risk for end-stage renal disease. They suggested that AKI accelerates the progression of renal disease. Coca and colleagues^18^ found in a meta-analysis of cardiac surgery patients that AKI was associated with a nine-fold increase in the risk of long-term CKD development. However, the current study by Bille and colleagues provides more granular detail through its systematic classification of baseline CKD stages, use of rigorous KDIGO-defined progression endpoints, competing-risk methodology accounting for mortality, and extended 5-yr follow-up with comprehensive laboratory data.

Subsequent research needs to align epidemiological insight with actionable intervention. Large, multicentre randomised trials will need to determine whether postoperative kidney monitoring and early initiation of renoprotective measures can mitigate CKD development in at-risk patients. Mechanistic studies combining biomarker and imaging data could clarify the translation of acute perioperative injuries to irreversible nephron loss and fibrosis. Finally, implementation studies will need to determine the feasibility, cost-effectiveness, and patient-centred benefit of multidisciplinary ‘renal recovery’ programs after cardiac surgery. Hopefully, the future will show whether perioperative AKI and progression of CKD are modifiable outcomes determining long-term cardiovascular and renal health.

## Authors’ contributions

Writing—original draft: both authors

## Funding

The European Society of Anaesthesiology and Intensive Care and BJA Charity (BJA-ESAIC_GR_2025_AH to AHH); the Netherlands Organisation for Health Research and Development (Veni-09150162410006 to AHH).
